# Screening of Substance Abuse Among the General Population in Saudi Arabia: A Cross-Sectional Study

**DOI:** 10.7759/cureus.84164

**Published:** 2025-05-15

**Authors:** Ayat ElZyat, Sadia Sultan, Aminah Altakroni, Ghyidaa Khan, Layan Alharbi, Alaa Magdy

**Affiliations:** 1 Clinical Sciences, Fakeeh College for Medical Sciences, Jeddah, SAU; 2 Medical Student, Bachelor of Medicine and Surgery Program, Fakeeh College for Medical sciences, Jeddah, SAU

**Keywords:** dast-10, diagnostic and statistical manual of mental disorders dsm-v, drug abuse, drug addiction, early detection

## Abstract

Background

Substance abuse refers to the harmful or hazardous use of psychoactive substances. Substance abuse is a pattern of problematic use that causes substantial impairment or distress, such as engaging in risky use or failing to meet important duties.

Methods

A cross-sectional study was conducted in Jeddah, Saudi Arabia, from 1^st^ January 2024 to 1^st^ April 2024, to assess the prevalence of substance abuse and its associated factors among 360 participants of the general population. The validated Arabic version of the Drug Abuse Screening Test-10 (DAST-10) by a confirmatory factor analysis (CFA) was used to assess the prevalence of substance abuse.

Results

The DAST-10 scale showed that the majority of participants reported no problems (56%), over one-third reported low-level problems (35%), while only 6% and 3% reported moderate and substantial levels, respectively. The multivariate regression analysis to predict substance abuse among the studied participants showed that smoking and having a close friend with substance abuse positively affect substance abuse, while sharing life aspects with the family is negatively associated with substance abuse.

Conclusion

Substance abuse was prevalent at low, moderate, and substantial levels in the general population of Saudi Arabia (total 44%). Substance abuse was more prevalent among the male population. Specific risk factors such as smoking and peer influence in the form of having a close friend with substance abuse were significantly contributing to substance abuse (P-value <0.05). These findings align with research indicating that familial factors, such as childhood maltreatment, parental substance abuse, and family socioeconomic status, contribute to increased substance use.

## Introduction

Substance abuse has been defined by the Diagnostic and Statistical Manual of Mental Disorders (DSM-5) as a pattern of problematic use of a substance that leads to clinically significant impairment or agony [1]. The fundamental criteria for substance abuse according to DSM-5 include: intermittent use of the substance leading to a failure to perform major duties at work, school, or home; intermittent use of the substance in situations where it is physically dangerous (e.g., driving while intoxicated); intermittent substance-related legal problems; and continued drug usage despite persistent or sporadic interpersonal or social issues brought on by or made worse by the drug's effects [2].

The terms "use," "abuse," "dependence," and "addiction" are often used to describe different stages or levels of problematic substance use, though the precise definitions and distinctions can vary [3]. Substance use refers to the consumption or utilization of a substance, whether it is legal or illegal, without necessarily indicating any problematic patterns. According to the DSM, substance abuse is a pattern of problematic use that causes substantial impairment or distress, such as engaging in risky use or failing to meet important duties [4].

Substance dependence represents a more severe kind of substance use disorder, marked by tolerance (requiring increased amounts of the substance to attain the same benefit), withdrawal symptoms upon cessation, and a diminished capacity to regulate usage. Addiction is different from abuse as it includes both substance abuse and dependence. It is marked by compulsive, uncontrolled substance use notwithstanding adverse consequences [5]. In Saudi Arabia, it was reported that lifetime prevalence of any substance use disorders were 4.03% during the period from 2014 to 2016 [6].

Early intervention can lead to more effective treatment and a better chance of achieving long-term recovery. Spotting substance abuse early can help prevent or lessen the severity of harmful effects, including damage to organs, loss of employment, and issues in relationships [7]. Identifying and treating issues early can lessen the strain on the healthcare system by stopping the progression of substance use disorders and the related health issues. Early action allows people to keep healthier connections, educational or work paths, and overall health, leading to a higher life satisfaction [8].

Screening, which employs early detection, provides more economic benefits than managing advanced substance use disorders and is a successful strategy for identifying at-risk groups and tailoring interventions [9].​​​* *Early detection can guide prevention efforts, helping to pinpoint at-risk groups and customize solutions to tackle the underlying issues of substance abuse. Effective early detection strategies can involve regular checks in healthcare facilities, educational programs in schools, and outreach in the community [10]. Therefore, this study aims to determine the prevalence of substance abuse and its associated factors among the Saudi population. Our hypothesis states that sociodemographic and psychosocial elements such as smoking habits and peer influence have a significant effect on substance abuse rates.

## Materials and methods

Study design, setting, and participants

This cross-sectional study was conducted in Jeddah, Saudi Arabia, from 1st January 2025 to 1st April 2025, to assess the prevalence of substance abuse and its associated factors among the general population. Both Saudi and non-Saudi adults over the age of 18 years who lived in Jeddah met the inclusion requirements. Those with a history of recovery from substance abuse were excluded. Fakeeh Institutional Review Board (F-IRB) in Jeddah granted ethical approval to the study (approval number FIRB-24-0009).

Sample size and data collection

The sample size was calculated using the Epi-Info software (United States Centers for Disease Control and Prevention, Atlanta, USA), and 360 participants were included. The participants were recruited using volunteer sampling (self-selection sampling) technique, and the questionnaire was self-administered. This approach was appropriate for the sensitivity of the topic, as individuals may be hesitant to participate unless they feel safe and choose to opt in voluntarily. Also, it is logistically easier and more cost-effective to recruit participants who respond to an announcement on social media. The survey was distributed through various social media and messaging platforms, including Facebook, WhatsApp, Twitter (X), Instagram, and Snapchat.

Google Forms was used to distribute the survey. The first section explained the purpose of the study and assured the confidentiality of the data before asking for informed consent to participate. Participants were informed that their responses would remain anonymous and securely stored. Additionally, identifying information such as names or contact details was not allowed, and IP addresses were not tracked. They were notified that by completing the questionnaire, they provided their informed consent to take part in the study. To prevent any participant from entering more than one response, Google Forms was configured to accept only one response per participant. Responses with missing data were not considered in the final analysis.

Data collection tools

The questionnaire included the following three sections: 1. General characteristics of the studied patients: age, gender, marital status, education, occupation, nationality, and income per month; 2. Potential risk factors for substance abuse include smoking, having close friends or family members who abuse drugs, having parents or spouses keep an eye on the participant's behavior and behaviors, and revealing personal details with family; 3. The validated Arabic version of Drug Abuse Screening Test-10 (DAST-10): it is a self-report instrument designed to screen for potential substance abuse and related problems (excluding alcohol and tobacco) that was developed by the test author Dr. Harvey Skinner, York University, Toronto, Canada and by the Centre for Addiction and Mental Health, Toronto, Canada [11]. It consists of 10 yes/no questions that assess an individual's involvement with drugs over the past 12 months. It covers a wide range of drugs, including cannabis (such as marijuana and hashish), cocaine (both crack and powder forms), heroin, hallucinogens like LSD (lysergic acid diethylamide), PCP (phenylcyclohexyl piperidine), and ecstasy, as well as inhalants such as glue, paint thinners, and nitrous oxide. It also assesses the use of stimulants like methamphetamine, sedatives, and tranquilizers such as benzodiazepines, opioid painkillers (e.g., oxycodone and hydrocodone), prescription drugs taken without a medical reason, and various synthetic or club drugs. Each "yes" answer is scored as 1, and the total score ranges from 0 to 10, with higher scores indicating a greater likelihood of problematic drug use.

For use in Arabic-speaking populations, this study used the validated Arabic version of DAST-10, 2020, that was developed by Prof. Hussam Murad and colleagues, funded by the Scientific Endowment (WAQF) at King Abdulaziz University, Jeddah, Saudi Arabia, and piloted in the Saudi population. This version maintains the original scale’s reliability and validity. It has shown acceptable internal consistency (Cronbach’s alpha typically > 0.7) [12].

Data management and analysis

The data was analyzed using IBM SPSS software version 22 (IBM Corp., Armonk, USA). Descriptive statistics for categorical variables included frequencies and percentages, while numerical variables were presented by mean ± SD. The Kolmogorov-Smirnov test indicated that the distribution of DAST-10 scores was non-normal. The association of the general characteristics with risk factors for substance abuse using the DAST-10 score was tested by non-parametric tests, i.e., Mann-Whitney and Kruskal-Wallis. A multivariate regression analysis of the factors influencing substance abuse among the study participants was conducted. Statistical significance was defined as a P-value <0.05 with a 95% confidence level. Tables and figures were used to present the data.

## Results

The study included 360 participants from the general population. The mean age of participants was 30 years (±11 years), with the majority of them being female (77.2%), and most being single (63.1%), followed by married (33.9%). A significant proportion of the participants had a bachelor's degree (82.5%), while fewer participants had only a high school education (11.9%) or postgraduate degrees (5.6%). The largest occupational group was students (26.1%), followed by office employees (21.7%). The vast majority of participants were Saudi nationals (93.1%), and the income distribution showed that most participants had an above-average (31.7%) or high income (45.6%) (Table 1).

**Table 1:** General characteristics of the studied participants (n=360).

General characteristics		Frequency	Percent
Gender	Male	82	22.8
	Female	278	77.2
Marital status	Single	227	63.1
	Divorced	11	3.1
	Married	122	33.9
Education	High school	43	11.9
	Bachelor	297	82.5
	Post-graduate	20	5.6
Occupation	Student	94	26.1
	Office employer	78	21.7
	Field employer	32	8.9
	Worker	21	5.8
	Other	24	6.7
	Retired/ Not working	111	30.8
Nationality	Saudi	335	93.1
	Non-Saudi	25	6.9
Income per month	Low	31	8.6
	Below average	51	14.2
	Above average	114	31.7
	High	164	45.6

The majority of participants (61.1%) were non-smokers, and 13.9% of them had a close friend with substance abuse. A small percentage (1.1%) reported a father with substance abuse issues, while 8.3% had a sibling (brother, sister, or general sibling category) affected. While 28.3% of participants reported that their behavior was "always" monitored, 20.6% stated they were "never" monitored. Nearly half (48.1%) of participants "always" shared aspects of life with their family, and a small portion (3.1%) "never" did this (Table 2).

**Table 2:** Risk factors for substance abuse among the studied participants (n=360).

Risk factors		Frequency	Percent
Smoking	Non-smoker	220	61.1
	Ex-smoker	45	12.5
	Current smoker	95	26.4
Close friends suffering from drug abuse	No	310	86.1
	Yes	50	13.9
Family members suffering from drug abuse	No	326	90.6
	Father	4	1.1
	Brother or sister	21	5.8
	Children	9	2.5
Parents or spouse monitor behaviour and actions of the participant	Never	74	20.6
	Rarely	50	13.9
	Sometimes	62	17.2
	Often	72	20.0
	Always	102	28.3
Sharing aspects of life with family	Never	11	3.1
	Rarely	23	6.4
	Sometimes	48	13.3
	Often	105	29.2
	Always	173	48.1

**Figure 1:**
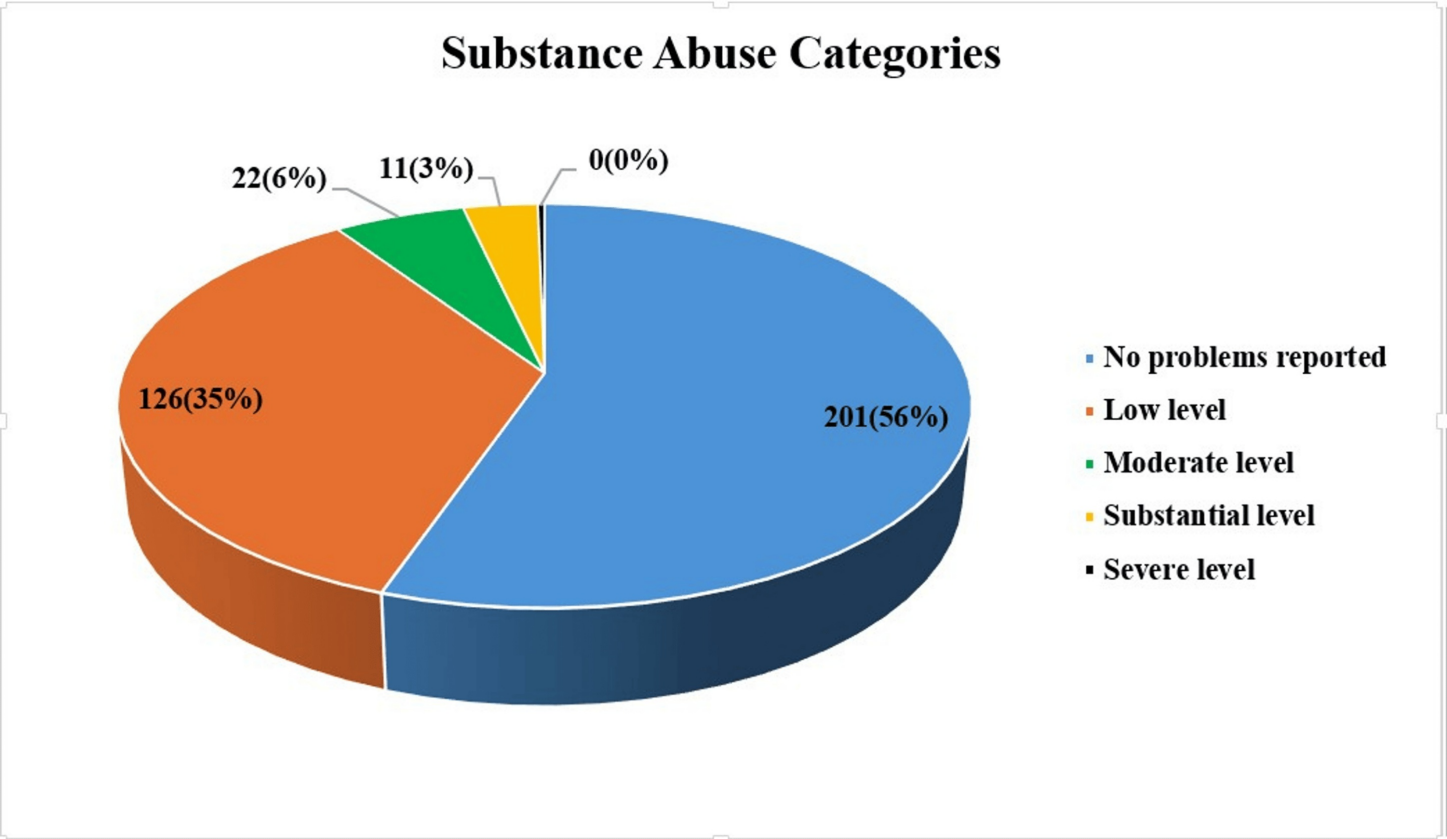
Screening for any substance abuse by DAST-10 among the studied participants (n=360). Categories are expressed as n(%); DAST-10: Drug Abuse Screening Test-10

The DAST-10 scale showed that the majority of participants were with “No problems reported” (56%), over one-third with “Low level" problems (35%), while only 6% and 3% with “Moderate” and “Substantial” levels, respectively (Figure 1).

The association between general characteristics and DAST-10 score showed a statistically significant higher score among the male population (P-value <0.05), while the other characteristics showed no statistically significant differences (P-value >0.05) (Table 3).

**Table 3:** The association between general characteristics and DAST-10 score among the studied population (n= 360). *P-value is statistically significant, Mann-Whitney test; ** P-value is not statistically significant, Kruskal-Wallis test; ***P-value is not statistically significant, Mann-Whitney test. DAST-10: Drug Abuse Screening Test-10

General characteristics		DAST-10 score (mean±SD)	P-value
Gender	Male	1.4±2	0.002*
	Female	0.7±1.3	
Marital status	Single	0.9±2	0.828**
	Divorced	0.8±1	
	Married	0.8±1	
Education	High school	0.9±2	0.981**
	Bachelor	0.9±2	
	Post-graduate	0.8±1	
Occupation	Student	0.8±1	0.475**
	Office employer	0.9±2	
	Field employer	1±2	
	Worker	0.9±2	
	Other	0.5±1	
	Retired/ Not working	0.9±2	
Nationality	Saudi	0.9±2	0.129***
	Non-Saudi	0.9±1	
Income per month	Low	0.9±2	0.871**
	Below average	0.9±2	
	Above average	0.7±1	
	High	0.9±2	

On the other hand, the association between risk factors for substance abuse and DAST-10 score showed a statistically significant higher score among current smokers and those who have friends with substance abuse (P-value <0.05), while the other factors showed no statistically significant differences (P-value >0.05) (Table 4). According to the results of the multivariate regression analysis used to predict substance misuse among the participants, greater DAST-10 scores were linked to smoking (B=0.238, P-value <0.05) and having a close friend who abuses drugs (B=1.087, P-value <0.05). On the other hand, DAST-10 scores were lower when family members shared aspects of their lives (B=-0.189, P-value <0.05) (Table 5).

**Table 4:** The association between risk factors for substance abuse and DAST-10 score among the studied population (n= 360). *P-value is statistically significant, Kruskal-Wallis test; ** P-value is statistically significant, Mann-Whitney test; ***P-value is not statistically significant, Kruskal-Wallis test. DAST-10: Drug Abuse Screening Test-10

Risk factors		DAST-10 score (mean±SD)	P-value
Smoking	Non-smoker	0.6±1	0.0001*
	Ex-smoker	1.1±2	
	Current smoker	1.5±2	
Close friends suffering from drug abuse	No	0.7±1	0.0001**
	Yes	2±3	
Family members suffering from drug abuse	No	0.8±1	0.688***
	Father	1±2	
	Brother or sister	1±2	
	Children	2±3	
Parents or spouse monitor behaviour and actions of the participant	Never	1±2	0.742***
	Rarely	0.7±1	
	Sometimes	1±2	
	Often	0.8±2	
	Always	0.8±1	
Sharing aspects of life with family	Never	1±2	0.529***
	Rarely	2±3	
	Sometimes	1±2	
	Often	0.7±1	
	Always	0.9±2	

**Table 5:** Multivariate regression analysis to predict substance abuse among the studied participants (n=360). *P-value is statistically significant; B represents unstandardized coefficients. β represents standardized coefficients. SA: substance abuse.

Independent variables	Unstandardized Coefficients		Standardized Coefficients	t	P-value	95.0% Confidence Interval for B	
	B	Std. Error	β			Lower Bound	Upper Bound
Age	.016	.010	.110	1.652	.099	-.003	.035
Sex	-.332	.217	-.089	-1.530	.127	-.760	.095
Education	.152	.200	.040	.758	.449	-.242	.545
Marital status	-.084	.071	-.076	-1.192	.234	-.224	.055
Occupation	-.069	.054	-.065	-1.267	.206	-.175	.038
Nationality	.164	.310	.027	.529	.597	-.446	.774
Income	-.024	.084	-.015	-.284	.777	-.190	.142
Smoking	.238	.101	.133	2.361	.019*	.040	.437
Friends with SA	1.087	.261	.241	4.174	.0001*	.575	1.600
Family member with SA	-.071	.098	-.042	-.718	.473	-.264	.123
Parents or spouse monitor behaviour and actions	-.002	.055	-.002	-.040	.968	-.110	.105
Sharing aspects of life with family	-.189	.077	-.129	-2.444	.015*	-.342	-.037

## Discussion

This cross-sectional study aimed to determine the prevalence of substance abuse among Saudi Arabia's general population using the screening approach. Furthermore, it examined the association between various sociodemographic and psychosocial factors with substance abuse. The Drug Abuse Screening Test (DAST-10) results revealed that the majority of participants (56%) reported no substance abuse concerns, while 35% exhibited low-level issues. Smaller proportions demonstrated moderate (6%) and substantial (3%) substance abuse problems. These findings suggest that while substance abuse is not widespread in the general population, a considerable percentage may be at risk, emphasizing the need for early intervention strategies. Previous studies have reported substance abuse prevalence rates of 7-8% [13], 9.4% [14], and 9.5% [15], aligning closely with our study findings.

Age was not a statistically significant predictor of substance abuse, suggesting that minor age variations within the studied population had little impact on the likelihood of substance use. Similarly, the 2010-2011 National Survey on Substance Use and Health estimated that 16.6% of the 25.1 million adolescents aged 12-17 years in the United States consumed alcohol or experimented with illicit drugs for the first time [16]. Male participants showed higher DAST-10 scores than females, which is consistent with previous studies suggesting a higher risk for substance abuse in males [17].

Education level, marital status, and occupation were also found to be non-significant predictors, suggesting that these variables may not independently influence substance use behaviors in this population. This finding was opposite to a previous study that reported that being single was more associated with substance abuse [18].

Nationality and income were not significant predictors of substance abuse, implying that economic status and national background did not play a substantial role in determining substance use risk among the participants. These findings highlight the potential universality of substance abuse risk factors across socioeconomic and cultural backgrounds, emphasizing the importance of psychosocial factors over economic constraints in shaping behaviors [19].

A substantial proportion of participants (61.1%) were non-smokers, while 26.4% were current smokers, and 12.5% were ex-smokers. The notable prevalence of current smokers suggests a possible association between smoking and substance use, as tobacco consumption is often recognized as a gateway to other forms of substance abuse. Research indicates that individuals with a history of cigarette smoking are significantly more likely to engage in the use of substances such as marijuana, cocaine, heroin, and crack. Notably, those who began smoking before the age of 15 were up to 80 times more likely to experiment with illegal drugs compared to non-smokers, with cocaine being the most frequently used substance among young cigarette smokers [20].

Furthermore, having friends who abuse substances was the strongest predictor of substance abuse (β = 0.241, p < 0.0001), while sharing life aspects with family (p = 0.015) was negatively associated. This underscores the critical role of peer influence in adolescent substance use, reinforcing the need for peer-based interventions and parental educational programs targeting at-risk youth. These findings suggest that peer influence is a major contributor to substance abuse, aligning with existing literature that highlights social networks as a critical factor in substance use initiation and continuation [15,18,21].

Conversely, strong family bonds, involvement, and open communication appear to serve as protective factors, which is in line with the study by Nawi et al. (2021) [22]. These findings are consistent with other research suggesting that familial risk factors, such as childhood maltreatment, parental substance abuse, marital status, parental education, parent-child relationships, family socioeconomic status, and parental approval of substance use, contribute to increased substance use [23].

Additionally, 8.3% had a sibling affected, and 1.1% had a father with substance abuse problems. These results highlight the influence of social circles and family on substance use behaviors. Furthermore, 28.3% of participants indicated that their behavior was "always" monitored by a parent or spouse, while 20.6% stated they were "never" monitored. Interestingly, parental or spousal monitoring of behavior was not significantly associated with substance abuse (β = -.002, p = .968), suggesting that supervision alone may not be an effective deterrent unless coupled with other protective measures [24].

Limitations

While this study provides valuable insights, it is subject to certain limitations. The cross-sectional design prevents causal inferences from being drawn. Additionally, self-reported data may be influenced by social desirability bias, potentially leading to underreporting of substance use. Expanding the sample size and incorporating qualitative methods could enhance the understanding of underlying motivations and contextual factors influencing substance abuse. One more limitation was the volunteer sampling technique, which imposes a selection bias. However, it is considered suitable for the sensitivity in collecting data on such a topic. Despite being a widely used tool to screen for substance abuse, DAST-10 still lacks the screening for specific substances, but it gives general information about substance abuse, which is suitable for the aim of the study.

Recommendations

Based on the findings of this study, initiatives in the form of national media campaigns and school-parent communication enhancement programs that strengthen parent-child communication, increase parental awareness, and encourage early monitoring of risky behaviors are recommended to be established. Moreover, it is recommended to develop educational programs that address the risks of substance use, focusing on peer pressure, smoking as a gateway behavior, and coping strategies for youth. Also, peer-support groups to be implemented and directed to those with substance abuse. Finally, there is an intense need to equip primary care providers with brief screening tools (such as DAST-10) to identify early signs of substance use, particularly in high-risk individuals.

Future research should further investigate long-term trends and the effectiveness of preventive measures in reducing substance abuse in Saudi Arabia. Also, research studies on vulnerable groups, such as adolescents and university students, are recommended. Qualitative studies using anonymized interview-based approaches to explore the potential factors that initiate the use of substances that impose the risk of abuse or addiction are recommended. Also, longitudinal studies to assess the associated factors will provide a stronger level of evidence.

## Conclusions

In conclusion, the findings indicate that substance abuse was prevalent at low, moderate, and substantial levels in the general population of Saudi Arabia. Substance abuse was more prevalent among the male population. Close peer associations and tobacco use significantly contribute to substance abuse, while family engagement plays a protective role. The study’s findings imply that substance abuse prevention efforts in Saudi Arabia should prioritize high-risk groups, particularly males, and address modifiable risk factors such as smoking and peer influence. Early intervention programs in schools and universities can help mitigate these risks by educating youth on the consequences of substance use and strengthening their ability to resist peer pressure. Public health campaigns should also raise awareness about how social environments contribute to risky behaviors. Additionally, the protective role of strong family relationships suggests that involving families in substance abuse prevention strategies can be highly effective. Promoting open family communication and encouraging shared activities may serve as a buffer against drug use. Policymakers should consider integrating family-based and culturally sensitive approaches into national mental health and addiction prevention frameworks to support long-term behavioral change.
